# Moving Equity into Practice: Evaluation of an Online Asynchronous Continuing Medical Education Program for Rheumatology Care

**DOI:** 10.1177/23821205261443553

**Published:** 2026-04-13

**Authors:** Elle Sauve, Glen Hazlewood, Emilie Pianarosa, Megan Thomas, Nicole Johnson, Aurore Fifi-Mah, Richard Henry, Therese Lane, Michael Kuluva, Cheryl Koehn, Kelly English, Nejat Hassen, Tessa Kleissen, Diane Lacaille, Cheryl Barnabe

**Affiliations:** 1 Faculty of Science, 2129 University of Calgary, Calgary, AB, Canada; 2 Department of Medicine, 2129 Cumming School of Medicine, University of Calgary, Calgary, AB, Canada; 3 Department of Community Health Sciences, 2129 Cumming School of Medicine, University of Calgary, Calgary, AB, Canada; 4 McCaig Institute for Bone and Joint Health, 2129 University of Calgary, Calgary, AB, Canada; 5 258853 Arthritis Research Canada, Calgary, AB, Canada; 6 Cumming School of Medicine, 70401 University of Calgary, Calgary, AB, Canada; 7 Faculty of Pharmaceutical Sciences, 8166 University of British Columbia, Vancouver, British Columbia, Canada; 8 Department of Pediatrics, 70401 Cumming School of Medicine, University of Calgary, Calgary, AB, Canada; 9 Department of Psychiatry, Faculty of Medicine, 12367 McGill University, Montreal, Quebec, Canada; 10 Canadian Arthritis Patient Alliance, Canada; 11 Arthritis Consumer Experts, Vancouver, British Columbia, Canada; 12 Department of Physical Therapy, 8166 University of British Columbia, Arthritis Research Canada, Vancouver, British Columbia, Canada; 13 Department of Medicine, Faculty of Medicine, 8166 University of British Columbia, Vancouver, British Columbia, Canada

**Keywords:** Rheumatology, equity, continuing medical education

## Abstract

**Background:**

Persistent health inequities exist in the diagnosis, treatment, and outcomes of rheumatologic diseases among at-risk populations in Canada due to structural discrimination, provider bias, and limited guidance on equitable treatment. Prior research identified barriers and facilitators to rheumatology care access and proposed multi-level equity solutions. This informed the development of a 10-part, 3-module asynchronous online continuing medical education (CME) program for rheumatology clinicians. This study evaluates the program's acceptability and its impact on intended changes in provider practice.

**Methods:**

The CME was informed by input from seven communities at-risk for inequitable RA care and evidence-based CME practices. Modules address equity knowledge, community-specific challenges, and strategies for delivering equitable services. Offered nationally to Canadian Rheumatology Association members, the program evaluation assessed satisfaction, knowledge gains, and planned practice changes using descriptive statistics and thematic analysis.

**Results:**

Forty-six participants enrolled, with nearly half completing the certification. High satisfaction was reported, with 87% indicating increased awareness and an enhanced ability to support equity in practice. Intended changes included enhancing accessibility to care, implementing trauma-informed and culturally safe practices, and delivering equitable clinical care. One domain highlighted the importance of ongoing professional development and collaboration with local health and social service networks. Participants recommended improvements such as downloadable one-page summaries and case-based content.

**Conclusions:**

The four identified action domains offer concrete strategies to reduce disparities in care among underserved populations. Their strong endorsement by participants indicates high acceptability of the program. Participant feedback will support further refinement and advancement of this educational equity initiative.

## Introduction

Healthcare leaders and providers have been called to acknowledge and address the disparities experienced by specific population groups in accessing healthcare resources and the various challenges to equitable health outcomes they face. While undergraduate and postgraduate medical education learners will have exposure to this content through formal teaching as a requirement of program accreditation, there are few resources available for practitioners in practice. Further, available courses may not provide content specific to subspecialty care delivery, nor thoroughly explore the unique realities of various populations at risk for health inequities. Additional resources to gain knowledge and understanding about the sources of these disparities, and then learning how to employ supportive care approaches that promote health equity when interacting with persons from these populations, were identified as educational needs for rheumatology care teams in our country.

In prior years, a continuing professional development initiative focused on improving communication skills and relationship building approaches in the care of Indigenous patients was offered by the Canadian Rheumatology Association.^1^ During this same time period, the Canadian Rheumatology Association's Quality Care and Guideline Committees collaborated on an initiative to create guideline implementation recommendations for rheumatoid arthritis care. Our research team completed a thorough investigation to identify the barriers and facilitators experienced by specifically selected at-risk populations in Canada in accessing rheumatology care.^2,3^ This included rural and remote residents, Indigenous Peoples (in Canada, inclusive of First Nations, Metis and Inuit communities), the elderly with frailty, newcomers to Canada (first-generation immigrant and persons with refugee status), those of low socioeconomic status, persons with diversity in sexual orientation and gender identity and expression, people of reproductive age, and Black Canadian populations. Merging common experiences and solutions for these populations, we proposed five key strategies to address the realities of access to rheumatology care in Canada, specifically related to common population-based inequities.^2,4^ These strategies included actively improving the patient-practitioner relationship and increasing accessibility and coordination of care through alternative models. We also emphasized upholding autonomy in treatment selection while addressing logistical barriers and individualized therapy needs, collaborating with health supports valued by patients, and advocating for policy change and health system restructuring to ensure appropriate resource redistribution.

In order to support integration of these approaches into clinical practice, focusing on practical application and examples and the unique context of the rheumatology subspecialty, we partnered with expertise in online CME resource development. A 3 module, 10-part asynchronous online continuing medical education (CME) program was developed and deployed nationally to rheumatology clinicians. The objective of this analysis was to evaluate the engagement and acceptability of the e-learning strategy, receive feedback on areas for enhancement, and gain insight on the effectiveness of the program by documenting what intended changes in provider practice behaviors were reported after completing the CME program.

## Methods

### Place, Period and Nature of Study

The ADDIE (Analyze, Design, Develop, Implement, Evaluate) instructional design framework was applied in this work.^5^ All aspects of the research were conducted in Canada. Data for module content^2,3^ was collected from May 2018 to October 2022 and the modules were drafted and revised with planning committee feedback from November 2022 through to November 2023. The modules were available from January 2024 to July 2025 with continuous recruitment and intake. We conducted an evaluation of participant engagement, satisfaction with the course, and effectiveness.

### Module Content

Module content was based on original research focused on populations at-risk for inequities in RA care in Canada as described in the introduction. As described in previously published work,^2,3^ we conducted qualitative interviews with persons with lived experience and healthcare providers with experience in delivering care to members of these communities on 3 aspects: population-specific considerations for care, the barriers and challenges in accessing RA care, and considerations for RA therapy selection. We asked the interview participants to identify solutions to mitigate challenges, and ways for their provider to support their care. We additionally incorporated content suggested by patient partners in our research team, based on their lived experience with rheumatoid arthritis.

### Module Development

The CME course was hosted at the University of Calgary's Cumming School of Medicine by the Office of Continuing Medical Education and Professional Development (University of Calgary CME & PD). The Office is fully accredited by the Committee on Accreditation of Canadian Medical Education (CACME) and is an accredited CPD provider for The College of Family Physicians of Canada (CFPC) and the Royal College of Physicians and Surgeons of Canada (RCPSC). The ‘Equity in Rheumatology Care’ program was developed for online, asynchronous delivery. Evidence-based teaching strategies and best practices for online CME learning were employed in the program design guided by the education consultant provided by the Office. These included the use of timed modules, each ranging from 8 to 12 min in length, and incorporating interactive components and reflective activities such as multiple-choice questions and action-oriented case study examples throughout.^6–8^ A multimedia approach was used, combining video teaching delivered by specifically selected actors (to represent the communities of topic in the course) along with concise written sections. Module scripts, key messages and a short end-of-module knowledge quiz were drafted by one author (CB) and were then reviewed by the formal Planning Committee (required for national accreditation), the remainder of the research team, and members of the University of Calgary CME & PD office for suggested revisions prior to filming. Edge Communications Inc. was contracted as the production company, with filming of the English language modules in February 2023, and the French language models in September 2023. A Technical Developer then uploaded all content to a secure web portal. The research team members reviewed the modules as a whole prior to participant recruitment to confirm that all suggestions for revisions were incorporated and content was clear.

### Final Program

The final ‘Equity in Rheumatology’ CME program consisted of 3 modules with a total of 10 sections. The module objectives are summarized in Table 1. Module 1 was an introductory chapter focused on basic knowledge related to equity. Module 2 included 7 specific sections addressing the realities and challenges faced by each of the at-risk communities. Module 3 concluded the course with recommendations for equity-oriented practices and policy changes. Participants could elect to complete the entire course, or only modules pertaining to their needs and interests. Those seeking a certificate for section 3 professional development credits through the Royal College of Physicians and Surgeons of Canada were required to complete all modules, as well as provide written responses to cases for a minimum of 3 populations discussed in the program.

**Table 1.** Equity in Rheumatology Care Program Overview and Objectives.

CONTENT	OBJECTIVES
**COURSE OBJECTIVES**	Increase rheumatology health care provider awareness of inequities in rheumatology care. Provide guidance on effective practice models for treatment recommendation implementation at the patient, health services and health policy levels. Self-evaluation of practice through evidence-based reflection activities.
**MODULES**	
**MODULE 1**	Equity in rheumatology care Gain knowledge about equity, health equity, and intervention-generated inequity. Learn about sources of health inequity in Canadian society.
**MODULE 2** **2.1 RURAL AND REMOTE RESIDENTS** **2.2 INDIGENOUS PEOPLES** **2.3 ELDERLY WITH FRAILTY** **2.4 NEWCOMERS TO CANADA** **2.5 LOW SOCIOECONOMIC STATUS** **2.6 SEX AND GENDER** **2.7 BLACK CANADIANS**	Equity considerations for populations Describe inequities in rheumatology care in Canada. Identify solutions for challenges to accessing rheumatology care.
**MODULE 3**	Equity-oriented actions for rheumatology practice and policy Consider various options for treatment recommendation implementation to reduce health inequity at the patient, health services and health policy levels. Apply effective practice models for treatment recommendation implementation at the patient, health services and health policy levels using cases.

### Program Release & Participant Recruitment

Participants were recruited through the Canadian Rheumatology Association (CRA), and both fully licensed Canadian rheumatologists and rheumatology residents were eligible to participate in the study. Members of the Allied Health Professionals in Rheumatology Association (AHPA) could also request access. No exclusion criteria were applied. Members of these associations were offered the opportunity to participate in this program free of charge for 18 months, from January 2024 to July 2025, with initial course announcements sent out on 2 occasions in January and February 2024 as an e-blast. Sustained recruitment efforts included repeated promotion through association newsletters (monthly) and at both annual scientific meetings held during the recruitment period during the national update, and with course information on postcards distributed at the registration desk and on meeting room tables.

### Data Collection and Program Evaluation

It was optional for participants to complete the evaluation. In alignment with the Kirkpatrick Evaluation Framework,^9^ we evaluated domains of engagement, recording the number of members who initiated and completed the full program and those who documented self-reflection activities. Additionally, we requested participants to rate overall satisfaction with the program, content completeness, the quality of materials, program length, and relevance to practice indicate on a 5 option Likert scale (strongly agree, agree, neither agree or disagree, disagree, strongly disagree) used in all University of Calgary CME & PD evaluations as required by the national accreditation body. For evaluating effectiveness, we asked participants to self-rate their awareness of equity issues at the completion of the course. Participants were also asked to submit Commitment-to-Change Statements,^10^ serving to evaluate program effectiveness for the intended learning objectives, and including a self-rated level of commitment to implementing the proposed changes. Additionally, free text boxes were provided for participants to report what components of the program were most and least effective, suggestions for individual module and overall program improvement, and additional content that they felt should be included. The data collection tool is available as Supplementary File 1.

## Statistical Analysis

### Quantitative Data

Descriptive statistics were used to summarize participant demographic information, engagement, satisfaction, and gains in awareness.

Qualitative Data: Free-text content related to program effectiveness and suggestions for improvement were descriptively summarized. The content of the commitment-to-change statements was analyzed using a phenomenological thematic analysis model^11^ for exploring how healthcare providers would incorporate equitable care into their RA practice. This analysis includes a categorization technique to label data and group key concepts across all submitted commitment-to-change statements.

### Ethics

Ethical approval was provided by the University of Calgary Conjoint Health Research Ethics Board (REB 19-0695). All participants provided written informed consent at the time of registration for the course.

### Reporting Guidelines

The reporting of this study conforms to the SQUIRE Reporting Guidelines^12^ (see Supplementary File 2).

## Results

### Participants and Engagement

A total of 46 participants (*n* = 43 enrolled in the English-language modules, *n* = 3 enrolled in the French-language version) engaged with at least one module of the course, representing approximately 6% of the CRA membership. One third (37%) of participants completed all 10 sections, after which they were prompted to provide their demographics. The majority identified as practicing rheumatologists (73%), with an even distribution of years in medical practice (seven resident/early career, one mid career, six senior career), worked primarily in large metropolitan centers (>500 000 population) (67%), with few (<5) indicating they worked in rural and remote settings. Participants were primarily based in Ontario, with additional representation from Quebec, Alberta, Atlantic Canada, and Saskatchewan.

We received feedback on each module as follows: Module 1 (19 responses), Module 2 (13 responses), and Module 3 (13 responses).

### Satisfaction

**Figure 1.**
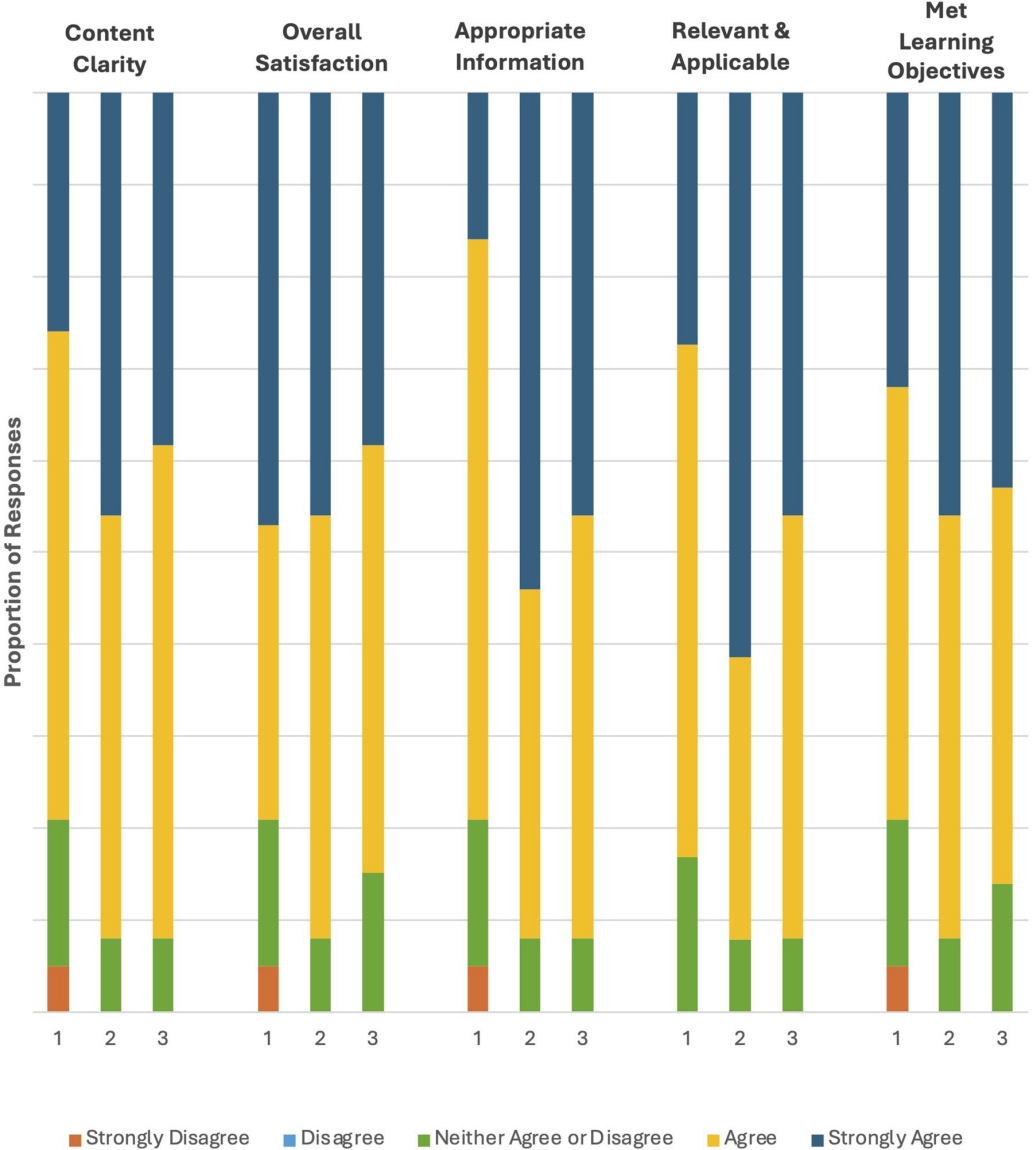
Individual module evaluation.

Overall participant satisfaction with the course modules was high (Figure 1). Module 1 was credited for its clarity, organization, and ability to meet all its original learning objectives. This included, upon completing the course, participants’ ability to recognize inequities in Canadian rheumatology care, apply effective models to support equitable treatment implementation across multiple levels, and engage in evidence-based self-reflection to evaluate and improve their practice. Modules 2 and 3 were also well-received, with over 90% of respondents indicating that the content was relevant and applicable to their clinical practice. Participants appreciated the inclusive, well-structured design of the modules and found the material engaging and informative. Module 3 stood out for its facilitation of active learning and high relevance to both equity and rheumatology practice.

### Awareness

**Figure 2.**
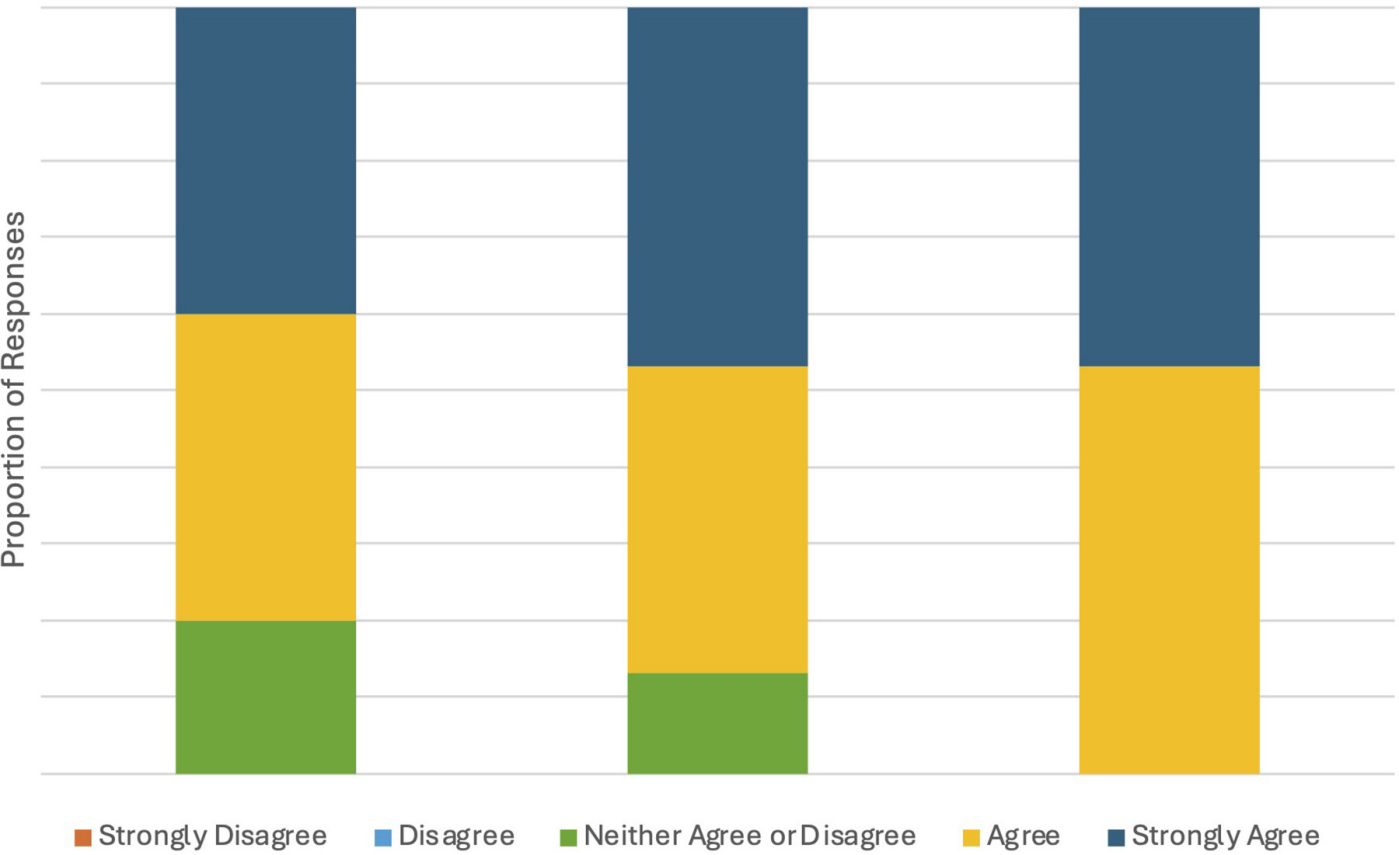
Program evaluation.

The course had a significant impact on participants’ awareness of equity issues in rheumatology care (Figure 2). Module 2, which explored the realities of 7 at-risk communities, helped participants connect course content directly to their clinical practices. Module 3 prompted meaningful reflection, with participants reporting a deeper understanding of how equity issues intersect with their work. Across the program, the majority of learners (87%) expressed increased confidence and awareness in supporting equitable healthcare delivery. Feedback consistently indicated that the modules enhance participants’ ability to recognize inequities and motivated them to apply more inclusive approaches in their professional settings.

### Participant Recommendations for Improving the Modules

Overall, participants expressed a preference for written content over embedded videos and recommended strengthening the trauma-informed components of the course to enhance its overall effectiveness. Feedback also proposed possible additions, such as integrating group discussion boards and creating a post-course summary page for easier future reference. Additional suggestions for improvement were to include more case examples, simplifying navigation within the course, and reducing content repetition.

### Commitment-to-Change Statements

**Figure 3.**
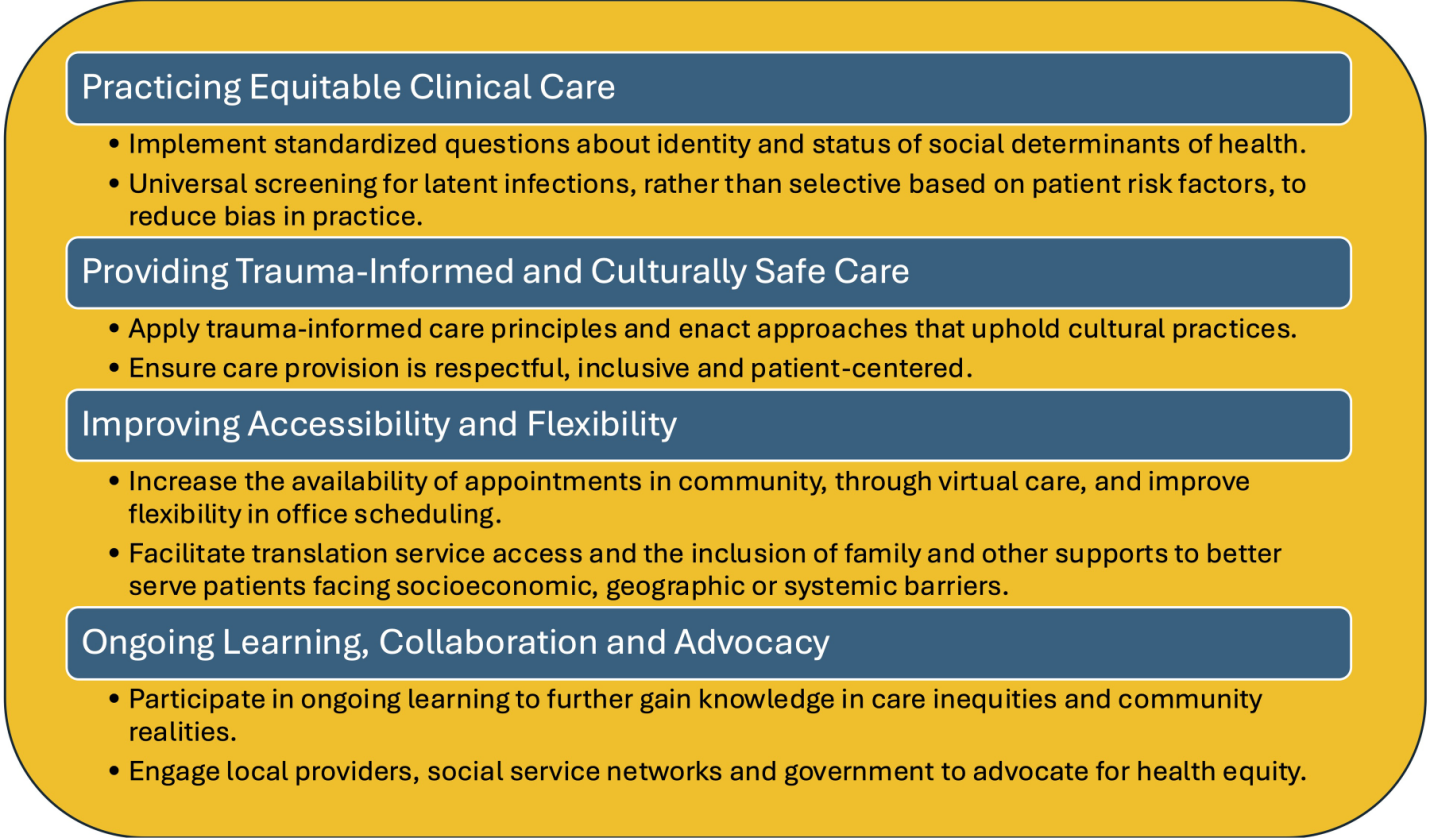
Thematic analysis of commitment to change statements.

Sixteen participants provided a total of 45 commitment-to-change statements. Four themes were identified from their intended actions: (1) Practicing Equitable Clinical Care, (2) Trauma-Informed and Culturally Safe Care, (3) Improving Accessibility and Flexibility, and (4) Ongoing Learning, Collaboration, and Advocacy (Figure 3).

- *Practicing Equitable Clinical Care:* Participants spoke about how they would embed approaches that would eliminate the potential for stereotyping. As an example, they indicated they would standardize identity and health screening questions to reduce the potential for biased questioning when interacting with members from at-risk groups.
- *Enhancing Trauma-Informed and Culturally Safe Care:* Participants were going to adjust their practice to now allow patients to have supportive people present throughout their visit to support the creation of a safe care environment. They indicated an intention to provide respectful, inclusive, and patient-centered care by integrating trauma-informed and culturally appropriate practices going forward.
- *Improving Accessibility and Flexibility:* Respondents were going to enact alternative models of care to increase accessibility, such as flexible appointment options and connecting patients with support services to overcome barriers related to language, location, and income.
- *Ongoing Learning, Collaboration, and Advocacy***:** The participants recognized their responsibility to expand their knowledge about health inequities and maintain open communication with primary care providers and health system leadership to promote more equitable care across Canada.

All participants (100%) expressed a high level of confidence in their ability to follow through on their commitment-to-change statements. Key barriers they foresaw included limited resources, time constraints, and challenges in adapting their communication styles to be more culturally appropriate. They identified potential solutions that included leveraging institutional support systems, improving care coordination, and engaging in further continuous professional development activities.

## Discussion

This study evaluated the engagement and acceptability of the online *Equity in Rheumatology Care* CME course and its impact on participants’ awareness and intended practice changes related to health equity in rheumatology care. The findings demonstrate high levels of participant satisfaction and self-reported increases in awareness and motivation to implement equity-oriented changes in clinical practice. Thematic analysis of the commitment-to-change statements revealed four themes that reflect meaningful intentions for integrating equitable care principles into rheumatology practice, aligning with the content of the program.

The course was positively received by those who completed it, with over 90% indicating that the content was relevant and applicable to their work. Modules were commended for their clarity, design, and practical relevance, particularly in fostering reflective practice and supporting the application of equity principles in daily clinical settings. Notably, Module 2, which addressed the lived experiences of at-risk populations, and Module 3, which encouraged action planning, was credited with significantly increasing awareness and confidence in applying equity-based approaches directly into practice.

The identification of the four themes – (1) Practicing Equitable Clinical Care, (2) Trauma-Informed and Culturally Safe Care, (3) Improving Accessibility and Flexibility, and (4) Ongoing Learning, Collaboration, and Advocacy—provided evidence that participants not only engaged with the course content but were also motivated to implement achievable changes in their practices. These intentions align with previously published strategies for addressing inequities in RA care in Canada.^2,4^ Participants also identified potential limitations that could affect their implementation process, while highlighting solutions that have been developed to overcome these limitations. This identification of potential challenges and solutions highlights possible future areas of study for supporting the implementation of more equitable care.

Virtual continuing medical education gained significant traction during the COVID-19 pandemic, with advantages including convenience and cost-effectiveness, while enhancing collaboration and introducing favorable learning formats to be effective at improving knowledge.^13^ Ours is one of few courses that promotes application of health equity principles in clinical practice. Other initiatives have included the Critical Dialogues for Action (CDFA) series, which is a monthly web-based seminar style activity for a general provider audience to support the integration of health equity and social accountability into care delivery.^14^ National and international thought leaders in health equity lead in-depth discussions with a focus on the practical application of equity principles in healthcare systems. Satisfaction, relevance and practical value with the series were high, attributed to the knowledge of the speakers and resources shared with attendees. Participants self-reported improved knowledge and indicated their intention to apply their learnings in educational and practice settings. Importantly, a shift from reflection to action was documented in this program's evaluation, with a gain in confidence to implement changes expressed. Sayani et al have shared their approach to co-design of an e-learning module aimed at building primary care providers’ capacity to delivery equity-oriented preventative care.^15^ Lung cancer screening is used as the exemplar, but with stated applicability of principles to diabetes care, immunization readiness, cervical cancer screening and substance use interventions. The e-module employs a variety of learning approaches including video narratives, case studies, a learner's notebook and interactive assessments, similar to our approach. Subsequent phases of their research will be to evaluate implementation and outcomes.

It is important to note limitations of this work. We did not set a target sample size a priori, but provided the course free of charge to encourage broad participation. Engagement with the content was low considering the national cohort of rheumatology care practitioners. While those who engaged with the program provided positive feedback, they may be self-selected for interest in the topic. Additionally, just one third elected to complete all 10 sections. This may be explained by the fact that participants were encouraged to complete the sections most relevant to their area of practice, rather than a requirement for every participant to complete the course in full. These points raise important considerations about resistance to learning about equity for practicing rheumatology care providers. Activities that embed this content in general CPD offerings in a systematic fashion or even requiring a minimum engagement in equity learnings for ongoing licensing requirements could increase uptake of the modules. Alternatively, it may reflect barriers to engagement in online CME, such as competing priorities and time constraints. We also acknowledge that the practitioner intentions in our research are purely by self-report. Future research will prioritize strategies for post-course follow-up to assess the longer-term impact on clinical practice and patient outcomes. This may include reaching out to participants—and, where appropriate, their patients—after a longer period to evaluate whether the course has led to sustained changes in clinical practice and patient care.

The next step is a second release of the course following the modifications suggested by participants, with the goal of making it a permanent educational resource for Canadian rheumatologists and providing practical and accessible knowledge across the country.

## Conclusions

This evaluation of the Equity in Rheumatology asynchronous CME program demonstrates its high acceptability among participants and its potential to influence clinical practice through enhanced awareness and commitment to equity-oriented care. The program successfully engaged learners in reflecting on structural inequities in rheumatology and supported them in formulating actionable intentions to address disparities, particularly for historically underserved populations.

However, the limited enrollment and completion rates underscore the need to address barriers to engagement in online CME formats. Time constraints and competing priorities likely contributed to the low uptake, despite the recognized importance of the topic. Additionally, the self-selection of participants may have introduced bias, with those already interested in health equity more likely to complete the course and provide positive feedback.

Next steps should include efforts to broaden the program's reach and impact. This may involve enhancing course interactivity and accessibility and possibly partnering with medical associations and regulatory bodies to explore opportunities for integrating equity-focused CME into mandatory or incentivized learning frameworks. Additional content to include additional populations and regional considerations, informed by ongoing feedback and lived experience engagement, will be important. Continued iteration and releases of the program are essential to embedding equity as a standard practice in rheumatology care across Canada.

## Supplemental Material

sj-docx-1-mde-10.1177_23821205261443553 - Supplemental material for Moving Equity into Practice: Evaluation of an Online Asynchronous Continuing Medical Education Program for Rheumatology Care Supplemental material, sj-docx-1-mde-10.1177_23821205261443553 for Moving Equity into Practice: Evaluation of an Online Asynchronous Continuing Medical Education Program for Rheumatology Care by Elle Sauve, Glen Hazlewood, Emilie Pianarosa, Megan Thomas, Nicole Johnson, Aurore Fifi-Mah, Richard Henry, Therese Lane, Michael Kuluva, Cheryl Koehn, Kelly English, Nejat Hassen, Tessa Kleissen, Diane Lacaille and Cheryl Barnabe in Journal of Medical Education and Curricular Development

sj-docx-2-mde-10.1177_23821205261443553 - Supplemental material for Moving Equity into Practice: Evaluation of an Online Asynchronous Continuing Medical Education Program for Rheumatology Care Supplemental material, sj-docx-2-mde-10.1177_23821205261443553 for Moving Equity into Practice: Evaluation of an Online Asynchronous Continuing Medical Education Program for Rheumatology Care by Elle Sauve, Glen Hazlewood, Emilie Pianarosa, Megan Thomas, Nicole Johnson, Aurore Fifi-Mah, Richard Henry, Therese Lane, Michael Kuluva, Cheryl Koehn, Kelly English, Nejat Hassen, Tessa Kleissen, Diane Lacaille and Cheryl Barnabe in Journal of Medical Education and Curricular Development
