# Supplementary material for: Moving Equity into Practice: Evaluation of an Online Asynchronous Continuing Medical Education Program for Rheumatology Care
Source: J Med Educ Curric Dev. 2026 Apr 13;13:23821205261443553. doi: 10.1177/23821205261443553 (PMC13080147; doi:10.1177/23821205261443553)
Supplement: sj-docx-1-mde-10.1177_23821205261443553 - Supplemental material for Moving Equity into Practice: Evaluation of an Online Asynchronous Continuing Medical Education Program for Rheumatology Care [file sj-docx-1-mde-10.1177_23821205261443553.docx]

**Equity in Rheumatology e-Learning Course**

**Evaluation Survey Questionnaire**

**Post-Module Completion 1, 2 and 3**

**Restate learning objectives <Piped fields/predefined text in Qualtrics>**

|  | Strongly Disagree | Neutral | Strongly Agree |
| --- | --- | --- | --- |
| a) The module met the stated learning objectives (1) | 1 2 3 4 5 | | |
| b) I learned something in this module that I will incorporate into my practice (2) | 1 2 3 4 5 | | |
| c) I found the module easy to navigate (3) | 1 2 3 4 5 | | |
| d) I found the content organized and easy to follow (4) | 1 2 3 4 5 | | |
| e) I found the amount of information provided to be appropriate to my learning needs (5) | 1 2 3 4 5 | | |
| f) I felt that the complexity of the information provided was appropriate to my learning needs (6) | 1 2 3 4 5 | | |
| g) I felt that the module included an appropriate balance between information and practice opportunities (7) | 1 2 3 4 5 | | |
| h) I felt that the module was inclusive and reflected diversity (8) | 1 2 3 4 5 | | |
| i) Potential conflicts of interest were clearly communicated (9) | 1 2 3 4 5 | | |
| j) Overall, I am satisfied with this module (10) | 1 2 3 4 5 | | |
| k) I would recommend this module to a colleague (11) | 1 2 3 4 5 | | |

**Did you perceive any degree of bias in any part of this module?**

- Yes [please specify] (3) __________________________________________________
- No (5)

**How could we improve this module?** *[free text]*

|  |
| --- |

**Immediately Post-Course**

Thank-you for completing this course. Your feedback is important for us further improving the course. The information collected through this survey will be used to evaluate the course. The information you share with us will be compiled with that of other participants for analytic and reporting purposes. Only aggregate results will be reported, and responses will not be linked to particular individuals. The collection of this information is authorized under Section 33(c) of the Freedom of Information and Protection of Privacy Act and the privacy protection provisions of that Act will protect it.

By clicking “next” you acknowledge that you understand the information above and indicate your consent to participate in this survey.

Q. **Your learning profile ID: ____**

Q. **Please rate your level of agreement with the statements below:**

|  | Strongly Disagree | Neutral | Strongly Agree |
| --- | --- | --- | --- |
| 1. The course content was relevant to the topic of equity in rheumatology. | 1 2 3 4 5 | | |
| 1. The course content was relevant to rheumatology practice. | 1 2 3 4 5 | | |
| 1. The course content was well organized. | 1 2 3 4 5 | | |
| 1. The course content was complete. | 1 2 3 4 5 | | |
| 1. The course content was effective in facilitating active learning. | 1 2 3 4 5 | | |
| 1. The course length was ideal. | 1 2 3 4 5 | | |
| 1. This course has increased my awareness about equity issues in rheumatology. | 1 2 3 4 5 | | |
| 1. This course has increased my ability to support equity in rheumatology practice. | 1 2 3 4 5 | | |
| 1. Overall, I was satisfied with this program. | 1 2 3 4 5 | | |

Q**. Did you perceive any degree of bias in any part of this program?**

□ Yes □ No
If YES, please comment: *[free text]*

**Q. Do you think there was content missing from the program? Yes No**

**If “Yes”, please describe:** *[free text]*

Q. **What was the most effective part of the program? Why?**

Q. **What was the least effective part of this program? Why?**

**Q. Please provide any other feedback on the program.** *[free text]*

**Q. How could we improve this program? [free text]**

**Q. (If you are not a physician, you may skip this question.)**

**Indicate which CanMEDS / CanMEDS-FM roles you felt were addressed during this educational activity. [Check all that apply]**

▢ Medical Expert / Family Medicine Expert

▢ Communicator

▢ Collaborator

▢ Leader

▢ Health Advocate

▢ Professional

▢ Scholar

**Q. Briefly describe the impact of this module on your practice.** *[free text]*

|  |
| --- |

**COMMITMENT TO CHANGE STATEMENTS***

List 3 changes you plan to make in the next 3 months related to this program’s focus, and your level of commitment to making each change:

|  | Level of Commitment to Change | | | |
| --- | --- | --- | --- | --- |
| Commitment Statement: | 1 (low) | 2 | 3 | 4 (high) |
| 1. |  |  |  |  |
| 2. |  |  |  |  |
| 3. |  |  |  |  |

What barriers might prevent you from applying what you've learned and what might help to overcome these barriers? *[free text]*

|  |
| --- |

DEMOGRAPHIC QUESTIONS

Q. **Please indicate your role*** Required

□ Rheumatologist

□ Advanced Clinician Practitioner in Arthritis Care (ACPAC)

□ Arthritis Allied Health Professional

□ CRA Member, non-clinical

□ Rheumatology Resident (specify: Adult, Pediatric)

□ Someone with lived arthritis experience, family member or friend

□ Prefer not to answer

Q. [If anything other than resident or patient selected ^] **Please indicate how long you have been in healthcare or medical practice.** * Required
□ 0-5 years
□ 6-15 years
□ >16 years
□ Prefer not to answer

Q. **Please indicate which province or region you primarily practice or reside in.**

[*List Or able to select (whole) province/territory on interactive map]*


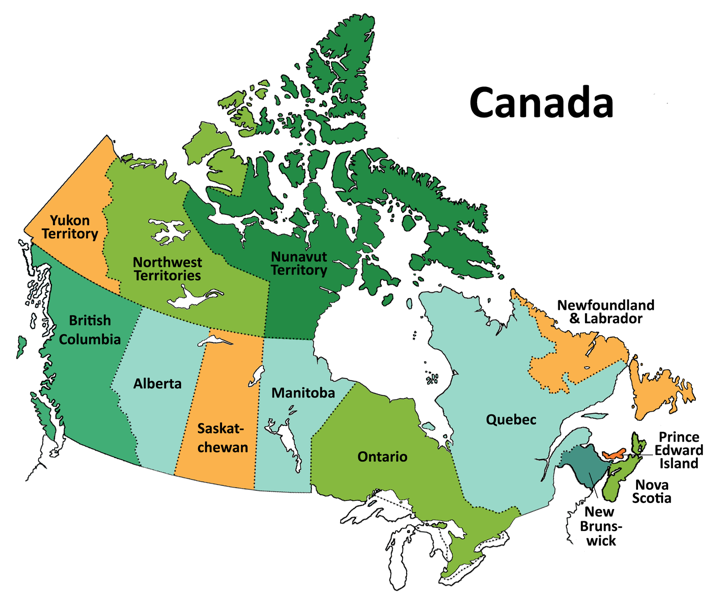


□ Prefer not to answer

Q. **[For HCP’s] Please indicate where your primary practice is located.**

□ Metro Centre (population > 500,000)

□ Urban (major urban centre with population greater than 25,000 but less than 500,000)

□ Large Rural Centre (population of 10,000 to less than 25,000)

□ Rural (population of less than 10,000 and up to 200km’s from a metro or urban centre)

□ Remote (greater than 200km’s from a metro or urban centre)

□ Prefer not to answer

Q. **[For HCP’s] Please indicate which other practice sites you work at, select all that apply.**

□ Metro Centre (population > 500,000)

□ Urban (major urban centre with population greater than 25,000 but less than 500,000)

□ Large Rural Centre (population of 10,000 to less than 25,000)

□ Rural (population of less than 10,000 and up to 200km’s from a metro or urban centre)

□ Remote (greater than 200km’s from a metro or urban centre)

□ Prefer not to answer

Q (suggestion): **What type of facility do you mainly work in? [Check all that apply]**

□ Acute Care

□ Community or Public Health Center

*Q: Are they facilities of interest specific to rheumatology that would be of interest to understand with the participants?*

Q. **How did you learn about this course?** * Required

[Please select all that apply]

□ CRA, AHPA or arthritis patient organization communication (newsletter, website, email, etc)

□ Online / web search

□ Colleague

□ Other [please specify]

□ Prefer not to answer

Q. **Have you completed any other health equity training prior to these modules?**

□ Yes

□ No

Q. **If yes** ^**, what type of training?**

[Please select all that apply]

□ Formal course in undergraduate degree prior to professional training

□ Lectures provided during professional training (medical school, residency, allied health professional training, graduate school)

□ Attended seminars or lectures

□ Attended a course

□ Reading or videos

□ Online training modules

□ Other (please specify) [For HCP’s] Please indicate which other practice sites you work at, select all that apply. *[free text]*

*We thank you for your time spent taking this survey. Your response has been recorded.*
